# Updated techniques and evidence for endoscopic ultrasound‐guided tissue acquisition from solid pancreatic lesions

**DOI:** 10.1002/deo2.399

**Published:** 2024-06-20

**Authors:** Masahiro Itonaga, Reiko Ashida, Masayuki Kitano

**Affiliations:** ^1^ Second Department of Internal Medicine Wakayama Medical University Wakayama Japan

**Keywords:** comprehensive genomic profiling, EUS‐TA, EUS‐FNA, needle tract seeding, pancreatic neoplasm

## Abstract

Endoscopic ultrasound‐guided tissue acquisition (EUS‐TA), including fine‐needle aspiration (EUS‐FNA) and fine‐needle biopsy (EUS‐FNB), has revolutionized specimen collection from intra‐abdominal organs, especially the pancreas. Advances in personalized medicine and more precise treatment have increased demands to collect specimens with higher cell counts, while preserving tissue structure, leading to the development of EUS‐FNB needles. EUS‐FNB has generally replaced EUS‐FNA as the procedure of choice for EUS‐TA of pancreatic cancer. Various techniques have been tested for their ability to enhance the diagnostic performance of EUS‐TA, including multiple methods of sampling at the time of puncture, on‐site specimen evaluation, and specimen processing. In addition, advances in next‐generation sequencing have made comprehensive genomic profiling of EUS‐TA samples feasible in routine clinical practice. The present review describes updates in EUS‐TA sampling techniques of pancreatic lesions, as well as methods for their evaluation.

## INTRODUCTION

Endoscopic ultrasound‐guided tissue acquisition (EUS‐TA), first described in 1992, has experienced significant advances,^1^, ^2^ showing greater sensitivity and specificity than other methods for diagnosing solid pancreatic lesions (SPLs). At present, EUS‐TA is considered a high‐priority method of choice for obtaining samples to diagnose SPLs.^3^, ^4^

Various factors can influence the diagnostic performance of EUS‐TA, such as needle shape/size, puncture technique, method of specimen collection, on‐site specimen evaluation, and specimen processing (Table 1). Although conventional EUS‐fine needle aspiration (EUS‐FNA) has been the method of choice for cytological diagnoses, this method is being replaced by EUS‐fine needle biopsy (EUS‐FNB), using “core needles” designed for histological diagnoses.^5^ The diagnostic accuracy of EUS‐FNB was shown to be equal to or greater than that of EUS‐FNA, as EUS‐FNB can obtain larger tissue volumes and require fewer punctures for diagnosis than EUS‐FNA.^6^, ^7^, ^8^ In addition, by obtaining larger tissue samples, EUS‐FNB enhances the ability to perform ancillary tests, including immunohistochemical assays and molecular profiling of tumor tissue.^9^

**TABLE 1:** Techniques and methods influencing the diagnostic performance of endoscopic ultrasound‐guided tissue acquisition.

Techniques and methods			Description
Puncture techniques	Door‐knocking method		Maximally rapid needle advancement within the target lesion after needle puncture, producing a loud knocking sound between the slider and the stopper.
	Fanning technique		To‐and‐fro movements of the needle, using the up/down knob of the endoscope during each pass. to direct the needle at different targets within the mass
	Torque technique		Twisting the body of the echoendoscope to the right (clockwise) or left (counterclockwise) without using the left/right control knob.
	Combined with contrast harmonic imaging		Identification of the appropriate puncture sites by distinguishing between viable, enhancing tumor tissue and non‐enhancing necrotic areas.
Specimen collection methods	No suction method		No negative pressure
	Suction method		Negative pressure is applied with a 10‐ or 20‐mL suction syringe connected to the needle.
	Slow‐pull method		Minimal negative pressure within the needle by slow removal of the stylet during sampling.
	Wet‐suction method		Negative pressure by filling the lumen of the needle with saline.
On‐site specimen evaluation	Rapid on‐site evaluation (ROSE)		Microscopic evaluation of Diff‐Quik stained cytological slides by a cytologist.
	Macroscopic on‐site evaluation (MOSE)		Gross inspection of specimens on a glass slide by an endoscopist.
	Visual on‐site evaluation (VOSE)		Gross inspection of specimens immersed in formalin by an endoscopist.
	Stereomicroscope on‐site evaluation (SOSE)		Stereoscopic evaluation of specimens in a Petri dish by an endoscopist.
	AI‐assisted ROSE or MOSE		Evaluation of images by AI
Specimen processing methods	Cytological diagnosis	Smear cytology (SC)	Cost‐effective, easy to use, and widely available
		Liquid‐based cytology (LBC)	An alternative to SC, due to its ability to prevent blood contamination and cell drying or depletion, which are known drawbacks of SC
	Histological diagnosis	Cell block	Method for evaluating histologic architecture and performing immunostaining and molecular assays through the collection of samples obtained with EUS‐FNA needles
		Core tissue	Collection with EUS‐FNB needles, routine processing into FFPE blocks, and easier handling compared with the cell block method.

Abbreviations: EUS‐FNA, EUS‐guided fine‐needle aspiration; EUS‐FNB, EUS‐guided fine‐needle biopsy; FFPE, formalin‐fixed paraffin‐embedded.

To increase the diagnostic performance of EUS‐TA, multiple puncture techniques, including door‐knocking, fanning, and torque methods, as well as combinations with contrast harmonic imaging, have been described.^10^ In addition, several tissue collection methods, including the suction, slow‐pull, and wet‐suction methods, have been reported.^10^ Several on‐site specimen evaluation methods were also developed to determine the appropriate timing for finishing EUS‐TA.^10^ Moreover, various methods have been developed for processing these pathological specimens, as processing can improve diagnostic performance.^11^

Advances in next‐generation sequencing have made comprehensive genomic profiling (CGP) feasible in routine clinical practice. Several methods have been described for effective CGP of EUS‐TA samples.^12^ Transgastric EUS‐TA, however, may also result in needle tract seeding (NTS), which can have deleterious effects in patients.^13^

Based on recent developments, the present review describes updated EUS‐TA sampling techniques, and evidence of their performance, for SPLs. In particular, this study describes technical aspects and novel applications for obtaining appropriate specimens during EUS‐TA.

### Puncture needles

**FIGURE 1:**
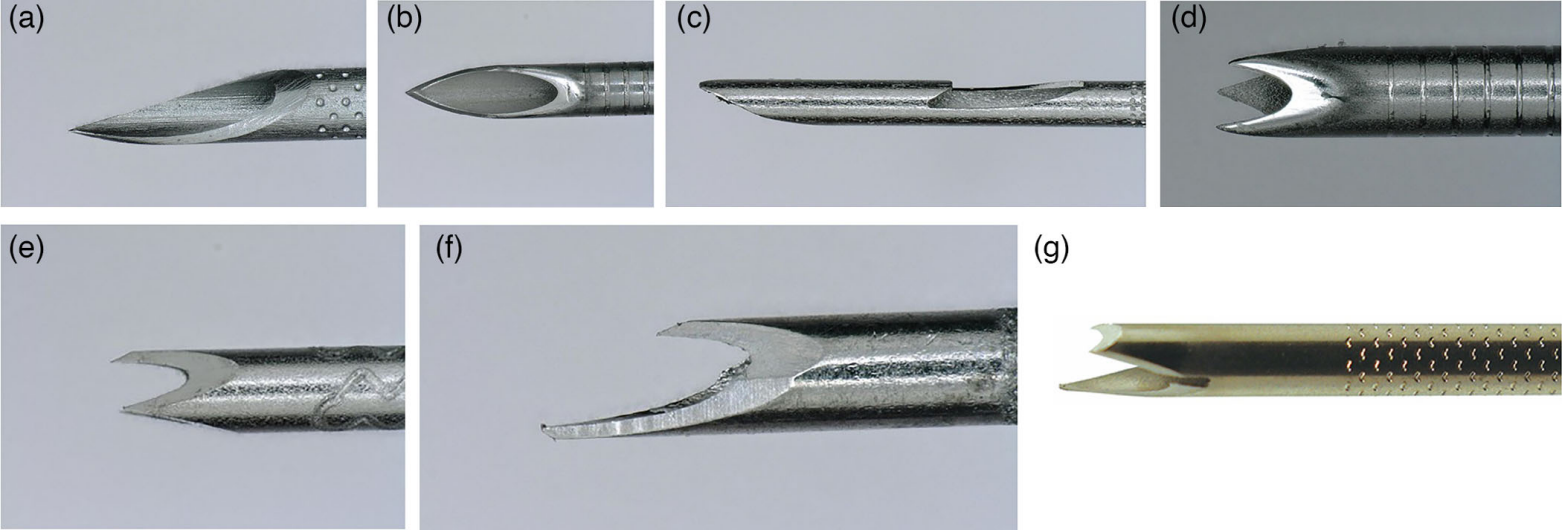
**Illustrations of needles designed for endoscopic ultrasound‐guided tissue acquisition**. (a) Menghini needle (EZ shot 3; Olympus Medical Systems). (b) Lancet needle (Expect; Boston Scientific). (c) Reverse‐beveled needle (ProCore; Cook Medical). (d) Franseen needle (Acquire; Boston Scientific). (e) Franseen needle (Sono‐Tip TopGain; Medico's Hirata). (f) Fork‐tip needle (SharkCore; Medtronic). (g) Multi‐blade three‐prong tip needle (Trident; MICRO‐TECH).

Puncture needles for EUS‐TA have been redesigned to collect larger sample volumes (Figure 1). Initially, needles such as Lancet and Menghini needles were developed for EUS‐FNA, followed by the development of core needles for EUS‐FNB, such as reverse bevel, antegrade core trap, fork‐tip, Franseen, and multi‐blade three‐prong tip needles. Many randomized clinical trials (RCTs) have compared EUS‐FNA with core needles in patients with SPLs^8^, ^14^, ^15^, ^16^, ^17^, ^18^, ^19^, ^20^, ^21^ (Table 2). Despite differences in study protocols, most of these trials reported that the diagnostic accuracy of EUS‐TA for SPLs using EUS‐FNA and EUS‐FNB needles was comparable. However, the quality of histological specimens obtained with core needles was superior.

**TABLE 2:** Randomized clinical trials comparing endoscopic ultrasound‐guided tissue acquisition using endoscopic ultrasound‐guided fine‐needle aspiration and endoscopic ultrasound‐guided fine‐needle biopsy needles in patients with solid pancreatic lesions.

First author (year)	Needles	Gauge of needles	Number of cases	Diagnostic accuracy		Quality of histological specimens
Bang (2012)	EUS‐FNA reverse‐bevel	22G 22G	56	100% 89.3%	*p* = 0.24	EUS‐FNA = reverse‐bevel
Lee (2014)	EUS‐FNA reverse‐bevel	22G and 25G 22G and 25G	192	94.8% 98.3%	*p* = 0.64	EUS‐FNA = reverse‐bevel
Alatawi (2015)	EUS‐FNA reverse‐bevel	22G 22G	100	88.4% 97.8%	*p* = 0.7	EUS‐FNA < reverse‐bevel
Kamata (2016)	EUS‐FNA reverse‐bevel	25G 25G	214	75.9% (per pass) 79.2% (per pass)	*p* = 0.56	EUS‐FNA < reverse‐bevel
Cheng (2018)	EUS‐FNA reverse‐bevel	22G 22G	377	80%^*^ 91.4%^*^	*p* = 0.001	EUS‐FNA < reverse‐bevel
Noh (2018)	EUS‐FNA reverse‐bevel	22G 22G	120	95.0% 93.3%	*p* = 0.56	EUS‐FNA = reverse‐bevel
Lee (2021)	EUS‐FNA Franseen	22G 22G	56	96.3% 86.2%	*p* = 0.35	EUS‐FNA< Franseen
Itonaga (2022)	EUS‐FNA Franseen	22G 22G	523	71.2% (per pass) 84% (per pass)	*p* < 0.001	EUS‐FNA < Franseen
Yoisri (2022)	EUS‐FNA Franseen	22G 22G	100	100% 100%	*p* = 1.0	EUS‐FNA< Franseen

Abbreviations: EUS‐FNA, endoscopic ultrasound‐guided fine needle aspiration; EUS‐FNB, EUS‐guided fine‐needle biopsy; EUS‐TA, EUS‐guided tissue acquisition; RCT, randomized clinical trial.

* Histologic diagnostic yield

Several RCTs have compared different types of core needles in patients with SPLs^22^, ^23^, ^24^, ^25^, ^26^ (Table 3). The diagnostic accuracy and the quality of histological specimens were superior when EUS‐TA was performed with Franseen needles than with antegrade core traps, as well as being superior when EUS‐TA was performed with Franseen and fork‐tip needles than with reverse‐bevel needles. By contrast, Franseen and fork‐tip needles showed comparable diagnostic accuracy histological specimen quality. Recently introduced FNB needles include Franseen‐shaped needles with three symmetrical heels and an added inner cut and needles with a multi‐blade, three‐prong tip, known as trident‐shaped needles. Compared with other FNB needles, these newly designed may improve puncture performance (See Figure 1).

**TABLE 3:** Randomized clinical trials comparing endoscopic ultrasound‐guided tissue acquisition using different types of core needles in patients with solid pancreatic lesions.

First author (year)	Needles	Gauge of needles	Number of cases	Diagnostic accuracy		Quality of histological specimens
Karsenti (2020)	Franseen Antegrade core trap	22G 20G	60	87% (per pass) 67% (per pass)	*p* = 0.02	Franseen > antegrade core trap
Crino (2020)	Fork‐tip Reverse‐bevel	22G and 25G 22G and 25G	192	92.7% 91.7%	*p* = 1.00	Fork‐tip > reverse‐bevel
Bang (2018)	Franseen Fork‐tip	22G 22G	100	94.0% 98.0%	*p* = 0.31	Franseen = fork‐tip
Bang (2021)	Reverse‐bevel Franseen Fork‐tip	22G 22G 22G	129	67.7% 92.7% 96.8%	*p* < 0.001	Reverse‐bevel < Franseen = fork‐tip
Ashat (2021)	Franseen Fork‐tip	22G 22G	150	86.7% 92%	*p* = 0.43	Franseen = fork‐tip

Abbreviations: EUS‐TA, endoscopic ultrasound‐guided tissue acquisition; RCT, randomized clinical trial.

Needle size may also affect sample volume and diagnostic accuracy. Three meta‐analyses have compared 25G and 22G EUS‐FNA needles, with one finding that the use of 25G needles improved diagnostic sensitivity, with similar specificity, whereas the other two reported no significant differences. Although these findings suggested that both 25G and 22G EUS‐FNA needles can be used to sample SPLs,^27^, ^28^, ^29^ the smaller 25G needle may provide a technical advantage over larger size needles when sampling highly fibrous, solid lesions. In addition, smaller needles may show greater maneuverability, particularly when sampling pancreatic head masses through the duodenum, due to their enhanced flexibility.^30^ An RCT comparing 25G and 22G EUS‐FNB needles in patients with SPLs found that their accuracy for detecting malignancy, as well as sample adequacy and quality, did not differ significantly.^31^ Although an RCT comparing 19G and 22G EUS‐FNA needles in patients with SPLs also found no significant difference in diagnostic accuracy, a secondary analysis limited to technically successful cases found that 19G EUS‐FNA needles showed significantly higher diagnostic accuracy.^32^ These findings suggested that 19G EUS‐FNA needles may be more useful for puncturable lesions.

### Sampling methods at the time of puncture

Many tissue sampling techniques, including door‐knocking, fanning, and torque methods, have been reported to increase the diagnostic performance and tissue yield of EUS‐TA following a puncture (Table 1, Puncture techniques). A multicenter, prospective trial comparing the conventional and door‐knocking methods of tissue sampling, each performed with two passes, found that the door‐knocking method acquired larger amounts of tissue during transgastric puncture, although it did not improve the accuracy of histological diagnosis.^33^ A prospective comparative study, in which SPL specimens were alternately obtained using the standard suction and slow‐pull with fanning techniques, found that the use of the latter significantly improved diagnostic accuracy compared with the former (88% vs. 71%, *p* = 0.044).^34^ A randomized trial involving 160 patients undergoing EUS‐TA for SPL compared a novel torque technique using EUS‐FNB needles with fanning and standard techniques.^35^ This study found that median quality scores were significantly higher for histological samples obtained using the torque and fanning techniques than the standard technique (*p* < 0.001), with the torque technique showing high sensitivity (93.4%) and accuracy (94.3%). These findings indicated that this newly developed torque technique has the potential to acquire better‐quality samples and may enhance the ability to diagnose SPLs.

Contrast harmonic EUS can be used to guide sampling by distinguishing viable, enhancing tumor tissue from non‐enhancing necrotic areas. Although several studies reported that contrast harmonic EUS‐TA could improve diagnostic performance,^36^, ^37^, ^38^ two RCTs found that contrast harmonic and conventional EUS‐TA had a similar diagnostic performance for SPLs.^39^, ^40^ Routine contrast harmonic EUS‐TA may therefore be unnecessary; rather, it should be selectively considered for re‐biopsy when the initial EUS‐TA yields false‐negative results.

Various methods have been described for collecting tissue samples during puncture procedures. These include a technique lacking suction, called the no suction method; the suction method, in which negative pressure is applied with a syringe; the slow‐pull method, in which the stylet is slowly withdrawn; and the wet‐suction method, in which the needle lumen is filled with saline and negative pressure is applied (Table 1, specimen collection methods). Two RCTs for SPLs showed that, compared with the no‐suction method, the application of 10‐mL suction improved the sensitivity and accuracy for diagnosis of malignancy,^41^, ^42^ suggesting that the suction method is more suitable for EUS‐TA for SPLs than the no‐suction method. By contrast, a meta‐analysis involving seven RCTs with 475 patients found no significant difference in specimen collection rates between the slow‐pull and suction methods, although blood clot rates were higher using the suction than the slow‐pull method.^43^ A multicenter, randomized, crossover trial comparing the wet‐suction and slow‐pull methods found that both tissue integrity scores and blood clot rates were higher using the wet‐suction method, although the two methods showed similar diagnostic performance and tissue sampling adequacy.^44^ Heparinized wet‐suction methods during EUS‐TA of SPLs method yielded specimens with longer lengths of white tissue and reduced blood contamination.^45^ In addition, the amount of extracted DNA correlated positively with white tissue, suggesting that heparinized wet suction may allow for next‐generation sequencing in CGP tests. A recent RCT involving four types of 22G needles for SPLs found that the suction method was optimal using reverse bevel and EUS‐FNA needles, whereas the stylet slow‐pull method was optimal with Franseen and fork‐tip needles.^25^ Taken together, these results suggest that the optimal aspiration technique may be dependent on the type of needle used. Additional studies, however, are required to determine the optimal sampling method.

### On‐site specimen evaluation methods

Various evaluation methods have been used to assess the quality of EUS‐TA samples, with these methods playing a crucial role in determining the appropriate timing for completing EUS‐TA. Rapid on‐site evaluation (ROSE), in which stained slides are examined in the procedure room, is widely utilized for the immediate assessment of sample cytology. ROSE has been reported to significantly improve the sensitivity and accuracy of sample evaluation, as well as reduce the number of inadequate specimens and needle passes.^46^ Despite these benefits, ROSE is available at few facilities and does not necessarily reduce procedure time. Additionally, the introduction of EUS‐FNB needles that allow for the collection of larger‐sized specimens with fewer needle passes suggests that the use of ROSE to reduce needle passes may no longer be necessary.^47^, ^48^, ^49^, ^50^

Macroscopic on‐site evaluation (MOSE), in which the presence or absence of white core specimens is visually determined, has been reported to be a useful alternative to ROSE.^51^ An RCT compared EUS‐TA with a 19‐gauge FNA needle using MOSE with a conventional method for SPLs >2 cm, with completion for the MOSE group defined as the acquisition of a significant macroscopically visible core.^52^ This study found that the number of needle passes was significantly lower in the MOSE than in the conventional group (2 vs. 3), although their diagnostic accuracy was comparable (92.6% vs. 89.3%). These results suggest that MOSE is a practical alternative for obtaining adequate tissue samples in settings where ROSE is not available, particularly during EUS‐TA using a 19‐gauge FNA needle for SPLs. A meta‐analysis of 14 studies assessing the diagnostic efficacy of EUS‐TA using MOSE found that MOSE had a pooled diagnostic accuracy rate of 91.3%, a sensitivity rate of 91.5%, and a specificity rate of 98.9%. Subgroup analyses of specimens collected with EUS‐FNB needles showed similar accuracy (90.6%), sensitivity (91.5%), and specificity (98.2%) rates.^53^ This study concluded that MOSE provides excellent diagnostic performance for both EUS‐FNA and EUS‐FNB needles.

Various methods have also been developed to evaluate specimens obtained by EUS‐TA. Visual on‐site evaluation involves ejecting EUS‐TA specimens into a vial containing formalin and visually evaluating the presence of macroscopically visible core, with the latter graded based on features such as ‘single or multiple’, ‘long (>4 mm) or short (≤4 mm)’ and ‘white or red‐mixed’.^54^ Stereomicroscope on‐site evaluation involves the examination of EUS‐TA specimens under a stereomicroscope to identify a white core; this method has shown a sensitivity of 91.4% for evaluating cores ≥ 11 mm in length, highlighting the importance of core length for tissue diagnosis.^55^ These methods are valuable in assessing the histological adequacy of EUS‐TA specimens.

Less is known about the ability of artificial intelligence (AI) to evaluate EUS‐TA specimens. AI‐assisted ROSE has been used to evaluate 467 digitized images of Diff‐Quik‐stained slides, with these slides divided into training and internal validation sets.^56^ Each slide was classified as being either positive or negative for cancer cells. AI‐based ROSE showed sensitivity, specificity, and accuracy rates of 96.0%, 98.5%, and 97.7%, respectively, in the training set and 79.1%, 85.4%, and 83.4%, respectively, in the internal validation set.^56^ The performance of AI‐based MOSE was evaluated on SPL samples obtained by EUS‐TA using 22‐gauge FNB needles.^57^ These specimens were added to a Petri dish containing saline, and images of the solid components were captured using a stereomicroscope. In evaluating its diagnostic performance, AI‐based MOSE showed sensitivity, specificity, and accuracy rates of 85.8%, 55.2%, and 71.8%, respectively, similar to the diagnostic performance of endoscopist assessment, which had sensitivity, specificity, and accuracy rates of 88.9%, 47.1%, and 81.6%, respectively.^57^ The rapid development of AI may result in the integration of AI‐assisted ROSE and MOSE into clinical practice.

### Methods of specimen processing

Samples can be prepared for either cytological or histological analysis. Cytology evaluates cells collected in fluid or on slides, whereas histology evaluates tissue samples, which are frequently preserved in formalin and embedded in paraffin blocks. The latter technique is well‐suited for examining substantial fragments with stromal elements. Combined evaluation of cytology and histology has demonstrated greater sensitivity and accuracy rates than either cytology or histology alone, suggesting that combined evaluation may be beneficial in clinical practice.^58^

Cytological diagnosis in EUS‐TA typically involves the evaluation of smear cytology (SC) samples, in which the collected specimen is directly smeared onto a glass slide.^59^ This method is cost‐effective, easy to perform, and widely available at most institutions. EUS‐TA evaluation of SC samples in the diagnosis of SPLs has shown sensitivities ranging from 64% to 94%.^46^ These slides are usually stained with Papanicolaou stain, followed by fixing with 95% alcohol. If the slides are not fixed while still wet, the cells may not adhere to the slides, resulting in the loss of many cells during the fixation process.^60^

Cytological examinations may also involve the evaluation of liquid‐based cytology (LBC) specimens.^61^ In LBC, collected cells are suspended in a liquid medium, homogenized, and smeared onto a glass slide. This method has been found to prevent blood contamination and the drying or depletion of cells, which are known drawbacks of SC.^61^ A meta‐analysis comparing LBC and SC with ROSE for SPLs obtained via EUS‐TA found that SC had significantly higher sensitivity than LBC (90% vs. 60%; *p* < 0.05), suggesting that LBC might be less effective in facilities where ROSE is available.^62^ By contrast, work from our group showed that the combination of LBC plus SC had significantly higher diagnostic accuracy than SC alone (94.1% vs. 69.6%; *p* < 0.001), suggesting that the combination of LBC plus SC is superior to LBC alone or SC alone in the clinical evaluation of SPLs.^63^

Small specimens collected with EUS‐FNA needles are prepared for histological examination using the cell block technique.^64^ Paraffin blocks are suitable for evaluating histological architecture, immunostaining, and molecular studies. The cell block method, however, has several limitations, including the need for additional, time‐consuming tissue processing steps and problems associated with thin paraffin blocks that cannot be repeatedly sectioned. The development of EUS‐FNB needles, which allow core tissue to be obtained, has reduced the need for the cell block method.^65^

### Use of CGP

To effectively perform CGP on EUS‐TA samples, these samples must be handled properly and meet specific pathological criteria. DNA must be extracted from formalin‐fixed, paraffin‐embedded (FFPE) tumor samples and analyzed. The percentage of tumor‐containing cells, tissue area, and DNA content required for CGP vary depending on the number of target genes analyzed. Foundation One CDx (F1CDx), commonly used in clinical settings, requires multiple tissue samples to analyze more than 300 genes concurrently. According to F1CDx test guidelines, sample adequacy is determined based on the following criteria: (1) the sample must include either one FFPE block and one hematoxylin and eosin‐stained slide, or 10 unstained slides (positively charged and unbaked, with tissue thickness of 4–5 µm) and one hematoxylin and eosin‐stained slide; (2) the surface area of the FFPE specimen must be at least 25 mm^2^ (5 × 5 mm); and (3) the percentage of tumor nuclei must be at least 20%, calculated as the ratio of tumor cells to the total number of cells with nuclei.^12^, ^66^

Several studies have evaluated the needle type and gauge required for genomic testing of pancreatic cancer samples (Table 4). The success rate of CGP using EUS‐TA specimens has been reported to range from 42% to 100%.^66^ Moreover, tumor volume and tumor purity required for testing may vary depending on the type of CGP.

**TABLE 4:** Studies describing genomic testing of pancreatic cancer using endoscopic ultrasound‐guided tissue acquisition samples.

First author (year)	No. of patients (*n*)/sample (*n*)	Cancer type	Sample type	Needle type and size	Analysis method/gene panel (*n*)	Required tumor volume/required tumor purity	Success rate of sequencing analysis, *n* (%)
Rodriguez (2016)	23	PDAC (15) Pancreatitis (8)	Cytology	FNA 22G	RNA‐seq (85)	Required 100 ng RNA / N/A	17/23 (73.9)
Lowery (2017)	52	PDAC	FFPE	FNA 22G FNB 19–25G	MSK‐IMPACT (410)	50 ng of DNA/ N/A	N/A
Aguirre (2018)	6	PDAC	Frozen FFPE	FNA 25G FNB 22G	WES RNA‐seq	N/A/ ≥5% tumor cell (before enrichment)	N/A
Larson (2018)	72/76	PDAC (74) Others (2)	FFPE	FNA 19–25G FNB 19–25G	F1CDx (324)	N/A/ ≥20% tumor cell	FNA 3/7 (42.9) FNB 38/54 (70.4)
Hayashi (2018)	20	PDAC	FFPE	FNA 22G	CLHURC comprehensive cancer panel (160)	50 ng DNA / N/A	15/20 (75)
Dreyer (2019)	41	PDAC (36) Others (5)	Frozen FFPE	FNB 19–22G	WGS RNA‐seq TGS	>50 ng DNA/ ≥25% tumor cell	31/43 (72)
Elhanafi (2019)	167	PDAC	Cytology Cell block	FNA 22G FNB 22G	TGS (47)	N/A/ ≥10% tumor cell	FNA 97/145 (66.9) FNB 20/22 (90.9)
Ishizawa (2020)	26	PDAC	Cytology	FNA 22G FNB 22G	Ion AmpliSeq Comprehensive Cancer Panel (409)	20 ng DNA/ N/A	26/26 (100)
Park (2020)	190	PDAC	Frozen	FNA 19–25G FNB 19–25G	Cancer SCANTM v1 (83)	50 ng DNA/ ≥30% tumor cell	FNA 38/57 (66.7) FNB 71/133 (53.4)
Carrara (2021)	33	PDAC	Frozen	FNB 22G	AmpliSeq Comprehensive Panel v3 (161)	10 ng DNA/ N/A	32/33 (97)
Kandel (2021)	50/100	PDAC (37) Others (13)	Frozen	FNA 25G versus FNB 22G or 19G cross‐over	F1CDx (324)	Approximately 10 ng/ lL/ 20%–30% tumor cell	FNA 7/50 (14) FNB 39/50 (78)
Hisada (2022)	33	PDAC	FFPE	FNB 19G	NCCOP (126)	Tissue slicing area ≥5 mm^2^/ Tumor cell content ≥20%	21/33 (63.6%)
Ikeda (2023)	150/153	PDAC	FFPE	FNA 22G FNB 22G FNB 19G	NCCOP (126)	Tissue slicing area ≥5 mm^2^/ Tumor cell content ≥20%	FNA 22G 4/35 (11.4) FNB 22G 14/43 (32.6) FNB 19G 42/75 (56.0)
Okuno (2023)	109	PDAC	FFPE	FNA 22G FNB 22G FNB 19G	F1CDx (324) NCCOP (126)	Approximately 10 ng/ lL/ 20%–30% tumor cell Tissue slicing area ≥5 mm^2^/ Tumor cell content ≥20%	FNA 22G 5/15 (33.3) FNB 22G 23/43 (53.5) FNB 19G 29/40 (72.5)

Abbreviations: F1CDx, FoundationOne CDx; FFPE, formalin‐fixed paraffin‐embedded; FNA, fine‐needle aspiration; FNB, fine‐needle biopsy; N/A, not available; NCCOP, OncoGuide NCC Oncopanel System; PDAC, pancreatic ductal adenocarcinoma; RNA‐seq, RNA sequencing; TGS, targeted next‐generation sequencing; WES, whole‐exome sequencing; WGS, whole‐genome sequencing.

An evaluation of EUS‐TA tissue sampling for CGP found that adequate tissue samples were significantly more likely to be obtained with EUS‐FNB than with EUS‐FNA needles (90.9% vs. 66.9%, *p* = 0.02).^9^ In addition, multivariate analysis showed that FNB needles alone (odds ratio: 4.95, 95% confidence interval 1.11–22.05, *p* = 0.04) were a significant independent predictor of CGP success.^9^ A retrospective multivariate analysis of factors influencing the success of CGP analysis in 190 patients who underwent EUS‐TA for pancreatic cancer found that needle gauge was the only significant factor associated with the success of CGP analysis, with 25G needles having a significantly lower success than 19/22G needles (38.8% vs. 60.9%, *p* = 0.003).^67^ An analysis of 109 EUS‐TA samples found that 61 (56.0%) were adequate for CGP.^68^ Univariate analysis showed that needle gauge and type were significantly associated with sample adequacy, with 22G FNA, 22G FNB, and 19G FNB needles showing sample adequacy rates of 33.3% (5/15), 53.5% (23/43), and 72.5% (29/40), respectively (*p* = 0.022). The adequacy rate for 19G FNB samples did not differ significantly from that of surgical specimens (*p* = 0.375), suggesting that 19G FNB needles were the first choice for unresectable pancreatic cancer.

### Needle tract seeding

The needle tract for pancreatic head cancers is usually resected en bloc with the head of the pancreas during curative surgery, whereas the needle tract for lesions in the pancreatic body or tail is beyond the surgical resection margins. Thus, cancer cell seeding through the needle tract may occur more frequently when lesions in the pancreatic body or tail are removed via a transgastric approach than when lesions in the pancreatic head are removed via a transduodenal approach.^69^ A nationwide survey of 235 institutions throughout Japan demonstrated that the incidence rates of NTS were 0.330% after EUS‐TA of all primary pancreatic tumors and 0.409% after EUS‐TA of pancreatic cancers.^13^ In addition, NTS was observed in 0.857% of patients who underwent transgastric EUS‐TA, but in none of those who underwent transduodenal EUS‐TA. On the other hand, Park et al. reported that 528 patients with cancer in the distal part of the pancreas underwent distal pancreatectomy. Among them, 193 were treated with EUS‐TA, while 335 did not receive EUS‐TA. Although NTS was observed in only two cases, recurrence‐free survival (RFS) and overall survival (OS) between the groups were similar.^70^ Yane et al. also reported the median RFS or OS was not significantly different between the EUS‐FNA group and the non‐EUS‐FNA group, although six patients (3.4%) in the EUS‐FNA group were diagnosed as having NTS.^71^ Despite the rate of NTS after preoperative EUS‐TA of patients with cancers of the pancreatic body and tail being non‐negligible, these results suggest that preoperative EUS‐TA in these patients did not adversely affect their recurrence or OS rates. Patients undergoing EUS‐TA before distal pancreatectomy should be monitored following surgery by upper gastrointestinal endoscopy and computed tomography to identify NTS as soon as possible.

## CONCLUSIONS

This review describes updates in EUS‐TA techniques for SPLs and evidence of their efficacy. The shapes of needles have been modified to collect larger tissue specimens for EUS‐TA. MOSE is a simple and rapid specimen method for the histological evaluation of samples obtained with EUS‐FNB needles. The ability to collect multiple samples from individual patients has made CGP of EUS‐TA specimens. NTS, however, may occur when transgastric EUS‐TA is performed before distal pancreatectomy.

## CONFLICT OF INTEREST STATEMENT

Masayuki Kitano has received honoraria from Olympus Corporation for delivering lectures at conferences and grants from Boston Scientific, Zeon Medical Inc., and Medicos Hirata Inc. Masahiro Itonaga and Reiko Ashida declare no conflict of interest.

## PATIENT CONSENT STATEMENT

N/A
